# The Theory of Relationship Sabotage: A Preliminary Evaluation of Conceptual Models Expanding on Attachment and Goal-Orientation Frameworks

**DOI:** 10.3390/bs15081091

**Published:** 2025-08-12

**Authors:** Raquel Peel

**Affiliations:** 1 Queensland Centre for Domestic and Family Violence Research, Central Queensland University, Mackay, QLD 4740, Australia; r.peel@cqu.edu.au; 2 Discipline of Psychology, University of Notre Dame Australia, Sydney, NSW 2007, Australia

**Keywords:** relationship sabotage, attachment, goal orientation, defensiveness, trust difficulty, lack of relationship skills

## Abstract

Introduction: The current study proposed the best model to explain relationship sabotage by comparing three competing conceptual models, using attachment and goal-orientation frameworks. Up until now, the literature had not comprehensively defined and modelled which defensive strategies commonly lead to relationship sabotage. Methods: A sample of 436 participants was recruited for the current study. Analyses were conducted using structural equation modelling over two analytical steps. First, a series of confirmatory analyses were conducted to test how the predicted latent variables fit in one-congeneric models. Secondly, three full models were tested. Results: Results showed the best model for relationship sabotage is non-recursive, and it does involve reciprocal effects between insecure attachment styles, relationship factors (i.e., perceived relationship quality and perceived relationship stress), and defensive strategies commonly observed in relationship sabotage (i.e., defensiveness, trust difficulty, and lack of relationship skills). Conclusions: The best model for relationship sabotage is not linear. While insecure attachment can lead to relationship sabotage, sabotaging relationships can reinforce existing insecure attachment styles and/or establish new vulnerable styles. Further, defensive strategies can influence how people perceive quality and stress in their relationship, which means that individuals’ own attitudes and behaviours might be preventing them from starting and maintaining fulfilling intimate relationships.

## 1. Introduction

Attachment and goal-orientation frameworks explain how relationship views, goals, and strategies are influenced by individuals’ responses to stress. More specifically, Rusk and Rothbaum (2010) proposed a model to explain that stressful moments in a relationship will activate existing insecure attachment styles, leading individuals to process stress using insecure views, self-validation goals, and defensive strategies.

We know that under stress (and facing the risk of getting hurt), individuals will often respond by behaving in ways that validate their fears and insecurities by using defensive strategies. And moments of stress will also influence how individuals perceive the quality of their relationships (Randall & Bodenmann, 2017). This is exacerbated by the fact that regardless of what a partner might do to contribute to relationship stress, individuals’ perceptions of what is happening for them (cognitively and emotionally) have the most significance (Walker et al., 2024). Thus, how individuals face feeling vulnerable in relationships will influence their stress management (Totenhagen et al., 2022).

Fear-driven responses in interpersonal and relational dynamics are significant predictors of defensive strategies, such as those seen in individuals experiencing fear of intimacy or rejection sensitivity; that is because fear contributes to repeated patterns of relational difficulty, including avoidance behaviours, defensiveness, and difficulties trusting others (Leiderman, 2025). Additionally, we know that the persistent use of defensive strategies for the purpose of self-protection can lead individuals to a cycle of not starting intimate engagements or not being able to maintain long-term engagements. That is because defensive strategies can become self-defeating when guided by self-validation goals (Zuckerman & Tsai, 2005). This phenomenon is named relationship sabotage.

Previously, the literature did not offer a clear definition for relationship sabotage or the defensive strategies responsible for this phenomenon. Up until the author’s own and colleagues’ work (Peel & Caltabiano, 2020, 2021; Peel et al., 2019), which conceptualised and defined relationship sabotage, this was a term often acknowledged in the literature (as contributing to relationship dissatisfaction and dissolution; e.g., Slade, 2020), but not clearly defined and operationalised. In Slade’s (2020) discussion paper outlining how relationship sabotage is presented in the context of anxious and avoidant attachment, individuals who experience anxious attachment are described as using ineffective conflict resolution strategies, such as inducing guilt to indirectly express their hurt, being controlling, and expressing or implying distrust in their partner. In our study (Peel & Caltabiano, 2021), these behaviours were captured under partner attack, defensiveness, trust difficulty, and controlling tendency themes. The same discussion paper (Slade, 2020) described individuals with an avoidant attachment style as more likely to engage in relationship self-defeating behaviours, such as withdrawing from conflict, lack of communication, and low relational investment. In our study (Peel & Caltabiano, 2021), this was well captured in the partner withdrawal and lack of relationship skills themes.

The author’s own and colleagues’ work (Peel & Caltabiano, 2020; Peel et al., 2019) defined relationship sabotage as a pattern of self-defeating attitudes and behaviours in (and out of) relationships preventing individuals from entering or maintaining fulfilling intimate engagements. That is to say, relationship sabotage is persistent and represents a repeated observance of maladaptive relational attitudes and behaviours. Relationship sabotage is not a single interaction with a partner or a single moment in a relationship. In two previous studies (Peel & Caltabiano, 2020; Peel et al., 2019), we have outlined three ways relationship sabotage is commonly presented in relationships. Some individuals sabotage by not entering relationships. This is due to a belief that they are not worthy or that the relationship is not going to work. Others are stuck in a cycle of successfully initiating a relationship, yet being unable to maintain long-term engagements, and embarking on a path that appears to be a destined break-up. In this case, individuals are moving through relationships too quickly, searching for “the one” and making quick assessments of their romantic partners. While others sabotage by staying in their relationships long-term, despite being unsatisfied or unhappy. In this case, individuals have “checked out”, or have lost hope, and are no longer working on their issues, thus hindering their chances of relationship satisfaction.

Furthermore, the author’s own and colleague’s work (Peel & Caltabiano, 2021) identified three defensive strategies that significantly contribute to relationship sabotage: (1) defensiveness, defined as a counterattack attitude or behaviour when responding to a perceived threat; (2) trust difficulty, defined as having trouble trusting intimate partners or often feeling jealous of a partner’s attention to others; and (3) lack of relationship skills, defined as having limited insight or awareness into potentially destructive tendencies in relationships, often as a result of poor relationship role models or negative interactions and outcomes from previous relationships. These strategies were persistently observed to impede relationship success, support the withdrawal of effort in relationships, and justify relationship failure. In support, a recent study from Iran (Sadeghi et al., 2025), following the introduction of our scale to measure relationship sabotage (Peel & Caltabiano, 2021), has identified that across cultures relationship sabotage is a significant mediator of attachment styles and relationship quality, supporting the author’s own and colleague’s work in this area.

Notwithstanding, the literature does not comprehensively explain or offer a model to show the path from defensive strategies to relationship sabotage. Moreover, the model proposed by Rusk and Rothbaum (2010) is linear and does not account for the interaction of factors (and their context) that may also contribute to relationship sabotage, such as insecure attachment styles and perceived relationship quality. Accordingly, there is still a need for further theoretical development in this area. Thus, the current study aims to propose the best model to explain relationship sabotage by comparing three competing conceptual models, using attachment and goal-orientation frameworks.

### Current Study

An important practice gaining favour in structural equation modelling (SEM) involves testing competing models to inform the best paths between constructs (Worthington & Whittaker, 2006). Therefore, three conceptual models were tested in the current study. All models encompassed four latent variables: (1) demographic factors (i.e., age, gender, and sexual orientation); (2) relationship factors (i.e., relationship status, longest relationship duration, perceived relationship quality, and perceived relationship stress); (3) insecure attachment (i.e., anxious and avoidant attachment styles); and (4) relationship sabotage (i.e., defensiveness, trust difficulty, and lack of relationship skills).

**Figure 1:**
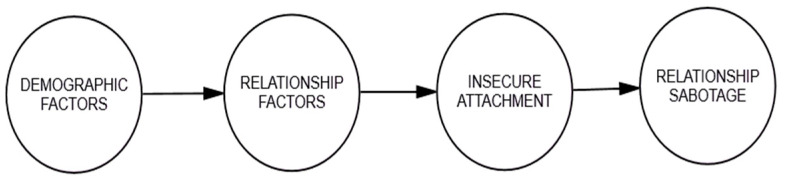
Conceptual Model 1 for relationship sabotage.

Conceptual Model 1 (see Figure 1) was drawn in accordance with Rusk and Rothbaum’s (2010) premise, which proposes a linear model showing that influenced by individuals’ demographic characteristics, stress in the relationship, and perceived relationship quality will activate the individual’s attachment style, and then, if insecurely attached, the individual is predicted to resort to self-defensive strategies to cope with the stressors in the relationship, leading to relationship sabotage.

**Figure 2:**
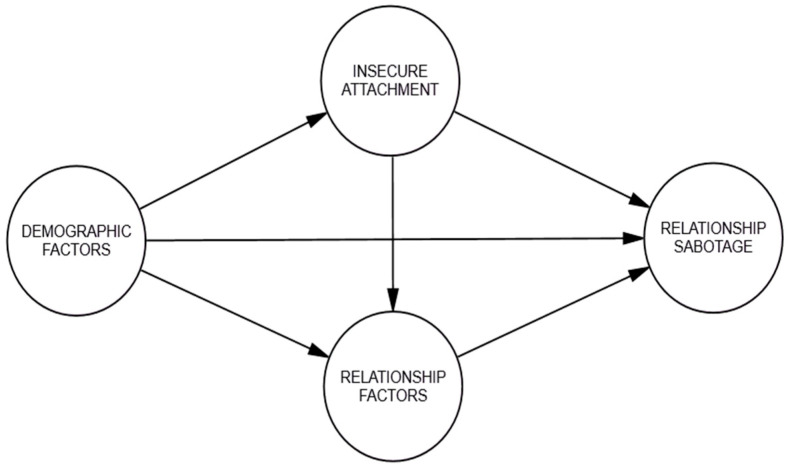
Conceptual Model 2 for relationship sabotage.

The next two models challenged Rusk and Rothbaum’s (2010) premise. Although it is agreed that relationship factors, such as stress and perceived relationship quality, can activate insecure attachment and, in turn, defensive responses, it is also possible that those responses are activated regardless of stress in the relationship and individuals’ demographic characteristics. Relationship sabotage is proposed to be a trait characteristic (i.e., stable but subject to change, the same way attachment is understood) learned and developed through life experiences. This means that relationship sabotage is most likely not a situational response dependent on stress or other relationship factors. Therefore, the next two models investigated the direct and indirect paths between relationship factors, insecure attachment, and relationship sabotage. Specifically, Conceptual Model 2 (see Figure 2) tested how relationship factors influence the relationship between insecure attachment and relationship sabotage.

**Figure 3:**
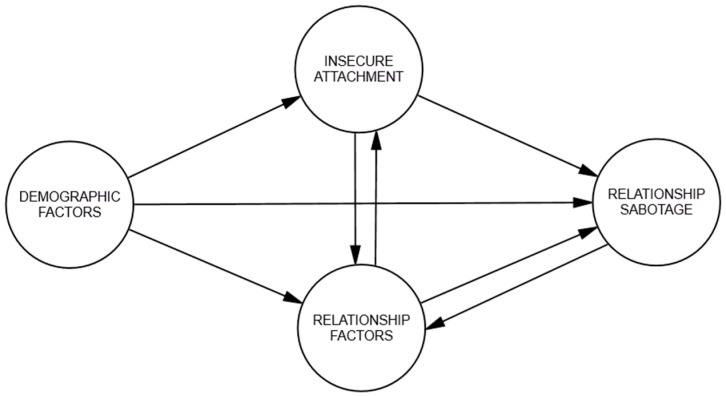
Conceptual Model 3 for relationship sabotage.

It is also important to note that Rusk and Rothbaum’s (2010) model assumed an existing attachment style that is activated in the presence of stress in the relationship. Conceptual Model 3 (see Figure 3) suggested that relationship factors and relationship sabotage influence existing attachment styles or develop new ones. Accordingly, the third model investigated how relationship sabotage influences the relationship between insecure attachment and relationship factors, and how insecure attachment influences the relationship between relationship factors and relationship sabotage.

## 2. Method

### 2.1. Participants and Procedure

The sample comprised 436 participants. Participants’ ages ranged between 14 and 75 years (*M* = 27.41, *SD* = 12.37). The distribution included 128 males (29.5%) and 302 females (69.5%), and 6 reported as ‘other’ (1%), with six descriptions for gender provided, including gender fluid (one), gender neutral (one), non-binary (one), queer (two), and transgender male (one). Regarding sexual orientation, most participants reported being heterosexual (336, 77%), while 74 (17%) self-identified as bisexual, 11 (2.5%) self-identified as homosexual, 8 (2%) reported as ‘other’, and 7 (1.5%) elected not to answer. For those who reported as ‘other’, eight provided descriptions for their sexuality, which included asexual (two), bi-curious (one), confused (one), panromantic and demisexual (one), pansexual (one), and questioning (two). A total of 250 (57%) participants reported being in a relationship (committed, de facto, or married), and 186 participants reported being single (43%). Further, participants reported a mean of 5.68 years (*SD* = 8.13, range 0 to 50) for their longest relationship duration. Ethics approval was obtained in accordance with the Declaration of Helsinki. Informed consent was obtained from all participants prior to them completing an online survey using Qualtrics. The data for the current study is a subset of a larger longitudinal project, which is still active (June, 2018–July, 2025). Data were analysed using AMOS 29 and SPSS 29 (IBM Statistics).

### 2.2. Measures

To reiterate, all models encompassed four latent variables, including the following observed variables: age, gender, and sexual orientation (i.e., demographic factors); relationship status, longest relationship duration, perceived relationship quality, and perceived relationship stress (i.e., relationship factors); anxious and avoidant attachment styles (i.e., insecure attachment); and defensiveness, trust difficulty, and lack of relationship skills (i.e., relationship sabotage). In particular, four measures are discussed in detail.

#### 2.2.1. Perceived Relationship Quality Components Inventory

The Perceived Relationship Quality Components Inventory Short-Form (PRQCI-SF; Fletcher et al., 2000) contains six components (1 item each) of perceived relationship quality: (1) satisfaction, (2) commitment, (3) intimacy, (4) trust, (5) passion, and (6) love. The original items were modified to include the word ‘current’ for individuals in a relationship and the word ‘previous’ for single individuals with relationship experience. An example of a modified satisfaction item is ‘How satisfied are you with your current relationship?’ or ‘How satisfied were you with your previous relationship?’. Items were modified to include responses from all individuals recruited. A five-point Likert scale, ranging from 1 (‘not at all’) to 5 (‘extremely’), was employed, where high scores indicated high levels of the measured dimensions. The overall score for perceived relationship quality was calculated by summing all six items. Therefore, scores ranging between 6 and 13 were considered low, 14 and 22 were moderate, and 23 and 30 were high. Regarding reliability, the instrument’s full version (18 items) showed good to excellent internal consistency for satisfaction (α = 0.93), commitment (α = 0.94), intimacy (α = 0.88), trust (α = 0.74), passion (α = 0.89), and love (α = 0.90). In addition, Fletcher et al. (2000) conducted CFA to compare alternative models for relationship quality to establish whether relationship quality is a mono-trait or multi-trait construct. The best-fitting model showed that all six perceived relationship quality factors represented a consistent indication of the individual’s general attitude towards their partner and the relationship, meaning that, although all factors were covariant, they also operated independently towards a single second-order factor. The current study also found good internal consistency for items referring to a current relationship (α = 0.86) and good internal consistency for items referring to a previous relationship (α = 0.89). Most participants (250, 57%) reported being in a relationship, which they rated as high quality overall (*M* = 22.99, *SD* = 5.69, range 6 to 30).

#### 2.2.2. Perceived Relationship Stress Scale

Perceived relationship stress was measured using an adapted version of Cohen et al.’s (1983) 14-item Perceived Stress Scale (PSS). The PSS was designed to measure the degree to which everyday situations in individuals’ lives are perceived as stressful. Items from the original scale were reworded to focus on stress in participants’ current or most recent relationship. The adapted measure was titled the Perceived Relationship Stress Scale (PRSS). For example, the item ‘In the last month, how often have you found that you could not cope with all the things that you had to do?’ was reworded as ‘How often have you found that you could not cope with all the stressors in your relationship?’. A five-point Likert scale, ranging from 1 (‘never’) to 5 (‘very often’), was employed, where high scores indicated high levels of the measured dimensions. The overall score for perceived relationship stress was calculated by summing all 10 items. Therefore, scores ranging between 10 and 22 were considered low, 23 and 37 were moderate, and 38 and 50 were high. Regarding reliability, Cohen et al. (1983) surveyed two samples of college students. In these samples, means of 23.18 (*SD* = 7.31, range 6 to 50) and 23.67 (*SD* = 7.79, range 5 to 44) were found. In addition, the 14-item scale showed good internal consistency, with alpha rates of 0.84 and 0.85. Good test–retest reliability was found for the samples of college students who were retested after two days (0.85). Regarding validity, the PSS was positively correlated to life-event impact scores in both samples (0.35 and 0.24; *p* < 0.05), showing convergent validity. The PSS was also used to measure stressful life events and showed good predictability of social anxiety in the two study samples (0.37 and 0.48, *p* < 0.001). In the current study, participants reported a mean of 27.52 (*SD* = 6.86, range 11 to 45), which was considered moderate. Moreover, the 10-item adapted scale for perceived relationship stress showed good internal consistency (α = 0.89).

#### 2.2.3. Experiences in Close Relationships Scale

The Experiences in Close Relationships Scale Short-Form (ECR-SF; Wei et al., 2007) assesses two insecure attachment dimensions (anxiety and avoidance) with 12 items (six items for each construct). An example of an anxiety dimension item is ‘I need a lot of reassurance that I am loved by my partner’, while an example of an avoidant dimension item is ‘I try to avoid getting too close to my partner’. A seven-point Likert scale, ranging from 1 (‘strongly disagree’) to 7 (‘strongly agree’), was employed, where high scores indicated high levels of the measured dimensions. Scores for anxious and avoidant attachment were calculated by summing even scores to compose anxious attachment and odd scores to compose avoidant attachment. Therefore, insecure attachment scores ranging between 6 and 17 were considered low, 18 and 30 were moderate, and 31 and 42 were high. In Wei et al.’s (2007) study, the instrument showed acceptable internal consistency for the anxiety subscale (α = 0.77) and the avoidance subscale (α = 0.78) and good test–retest reliability for both subscales (α = 0.82 and α = 0.89, respectively). Additionally, Lafontaine et al. (2016) conducted a study comparing all existing versions of the ECR and found the 12-item short version to have the best psychometric properties, with the anxiety subscale showing alpha values between 0.78 and 0.87 and the avoidance scale showing alpha values between 0.74 and 0.83. In the current study, the ECR-SF showed acceptable internal consistency for the anxiety subscale (α = 0.73), good internal consistency for the avoidance subscale (α = 0.80), and acceptable internal consistency for the total scale (α = 0.78). Participants in the current study scored a mean of 23.58 (*SD* = 6.86, range 6 to 41) for anxious attachment, which was considered moderate, and a mean of 16.11 (*SD* = 6.43, range 6 to 35) for avoidant attachment, which was considered low.

#### 2.2.4. Relationship Sabotage Scale

The Relationship Sabotage Scale (RSS; Peel & Caltabiano, 2021) contains 12 items and three subscales (4 items each): defensiveness, trust difficulty, and relationship skills. A seven-point Likert scale, ranging from 1 (‘strongly disagree’) to 7 (‘strongly agree’), is employed, where high scores indicate high levels of the measured dimensions. Once items are reverse coded, high scores in the relationship skills factor indicate a lack of relationship skills. An example of a defensiveness item is ‘I constantly feel criticised by my partner’, an example of a trust difficulty item is ‘I often get jealous of my partner’, and an example of a lack of relationship skills (reverse) item is “I am open to finding solutions and working out issues in the relationship.”

Internal reliability for the RSS was assessed with Cronbach’s alpha and coefficient *H*. In accordance with Hancock and Mueller (2001), scales developed using CFA are better assessed with coefficient *H*, as this measure provides a more robust way to evaluate latent measures created from observable construct indicators, such as regression coefficients, especially if items are not parallel. The Cronbach’s alpha calculation assumes that all items are parallel, which is often not the case, and is affected by the sign of the indicators’ loading. Alternatively, coefficient *H* is not limited by the strength and sign of items and draws information from all indicators (even from weaker variables) to reflect the construct. Further, Lord and Novick (1968) proposed that, if measures associated with a latent trait are congeneric, Cronbach’s alpha will be a lower-bound estimate of the true reliability. Therefore, both estimates are reported.

Using Cronbach’s alpha, the full set of items in the RSSS (12 items) indicated good internal consistency (α = 0.79). The sub-factors showed mostly acceptable to good reliability for defensiveness (α = 0.85), trust difficulty (α = 0.63), and relationship skills (α = 0.76). Using coefficient *H*, the full set of items indicated excellent internal consistency (*H* = 0.92). The sub-factors showed mostly acceptable to good reliability for defensiveness (*H* = 0.86), trust difficulty (*H* = 0.65), and relationship skills (*H* = 0.77). The RSSS has also been translated and replicated in Turkish samples, further supporting its cross-cultural applicability (Maden et al., 2023; Turan & Yildirim, 2023).

The scores for each relationship sabotage sub-factor were created using the factor score regression weights obtained from the one-factor congeneric measurement models, as recommended by Jöreskog (1967). This approach is unlike adding raw scores to represent subscales, which assumes that the items are parallel. Weighted composite variables best represent each variable’s unique contribution. Further, weighted composite variables are continuous, as opposed to Likert scale scores, which are ordinal. Therefore, for the purpose of creating weighted composite variables, factor score regression weights were rescaled to add up to a total of 1. Conclusively, relationship sabotage scores ranging between 1 and 3 were low, 4 was moderate, and 5 and 7 were high. For the current study, participants reported a mean of 2.88 (*SD* = 1.43, range 1 to 7) for defensiveness, 2.83 (*SD* = 1.19, range 1 to 6) for trust difficulty, and 2.06 (*SD* = 0.81, range 1 to 7) for relationship difficulty, which were all considered low.

### 2.3. Data Characteristics

The sample size was deemed adequate for the current study. This was assessed in accordance with the recommendations by Bentler and Chou (1987), Worthington and Whittaker (2006), and Kline (2016), which proposed a sample of a minimum of 200 participants and a minimum of 5:1 participants per parameter. As an example, the most complex model (Final Model 3) estimated 42 parameters (a ratio of 10:1). Data normality was assessed for the current study’s main variables. The perceived relationship quality data showed skewness values ranging from −0.96 to −1.67 and kurtosis values ranging from −0.09 to 2.80. The perceived relationship stress data showed skewness values ranging from 0.03 to 0.59 and kurtosis values ranging from −0.79 to 0.26. The attachment style data showed skewness values ranging from −0.59 to 1.81 and kurtosis values ranging from −1.35 to 4.32. The relationship sabotage data showed skewness values ranging from −0.05 to 1.74 and kurtosis values ranging from −1.43 to 5. Conclusively, the current study data showed mild deviations from normality and complied with the parameters recommended by Fabrigar et al. (1999) to treat the data as normally distributed (i.e., skewness < 2, kurtosis < 7). Finally, the current sample did not include missing data for the study variables.

### 2.4. Data Analysis

The current study followed two analytical steps. First, a series of Confirmatory Factor Analyses (CFAs) were conducted to test how the predicted latent variables fit in one-congeneric models. The one-congeneric model approach allows for factors of different weights within the same latent construct to contribute uniquely and does not assume that items are parallel. Second, three full models were compared. The full models were drawn and analysed using the six steps proposed by Bollen and Long (1993) and Kline (2016): (1) model conceptualisation involving three conceptual models; (2) path diagram construction and model specification, where paths between variables were drawn in accordance with the proposed hypotheses, as per Byrne’s (2010) recommendations; (3) model identification, which met the *t*-rule requirement, with the most complex model analysed in the current study (Final Model 3) showing 42 free parameters and 42 observable variables (i.e., 42 ≤ 42); (4) parameter estimation, using the Maximum Likelihood (ML) procedure, following the seminal work of Jöreskog (1967); (5) assessment of model fit, using six measures, including two fit statistics (chi-square [χ^2^] and root mean square error of approximation [RMSEA], and three incremental or comparative fit indices (goodness-of-fit index [GFI], comparative fit index [CFI], and Tucker–Lewis index [TLI]); and (6) model re-specification, informed by indices such as factor regression weights and error measurement and variance explained, to highlight the best alterations, with final alterations informed by the existing literature.

## 3. Results

### 3.1. Confirmatory Factor Analysis of Latent Variables

CFA was conducted for each of the four latent variables used in the full models (i.e., demographic factors, relationship factors, insecure attachment, and relationship sabotage).

#### 3.1.1. Demographic Factors

The latent variable for demographic factors was composed of age, gender, and sexual orientation. Model fit analysis indicated a good fit for this latent variable (*χ*^2^_(1)_ = 0.705, *p* = 0.401; RMSEA = 0.000 [0.000, 0.119], *p* = 0.610; GFI = 0.999; CFI = 1; TLI = 1; SRMR = 0.017).

#### 3.1.2. Relationship Factors

The latent variable for relationship factors was composed of relationship status, longest relationship duration, perceived relationship quality, and perceived relationship stress. Model fit analysis indicated a good fit for this latent variable (*χ*^2^_(1)_ = 0.885, *p* = 0.347; RMSEA = 0.000 [0.000, 0.124], *p* = 0.564; GFI = 0.999; CFI = 1; TLI = 1; SRMR = 0.011).

#### 3.1.3. Insecure Attachment

The latent variable for insecure attachment was composed of anxious attachment and avoidant attachment. Each sub-factor one-congeneric model fit separately for anxious and avoidant attachment (anxious attachment: *χ*^2^_(5)_ = 3.996, *p* = 0.550; RMSEA = 0.000 [0.000, 0.059], *p* = 0.901; GFI = 0.997; CFI = 1; TLI = 1; SRMR = 0.015; avoidant attachment: *χ*^2^_(6)_ = 7.843, *p* = 0.250; RMSEA = 0.027 [0.000, 0.071], *p* = 0.759; GFI = 0.994; CFI = 0.998; TLI = 0.995; SRMR = 0.013).

#### 3.1.4. Relationship Sabotage

The latent variable for relationship sabotage was composed of defensiveness, trust difficulty, and relationship skills. Model fit analysis indicated a good fit for this latent variable (*χ*^2^_(1)_ = 3.039, *p* = 0.081; RMSEA = 0.068 [0.000, 0.162], *p* = 0.244; GFI = 0.995; CFI = 0.986; TLI = 0.959; SRMR = 0.024), with the RMSEA fit statistic showing a partial good fit.

### 3.2. Full Structural Equation Models

Three full models were tested in accordance with Conceptual Models 1, 2, and 3.

#### 3.2.1. Model 1

**Figure 4:**
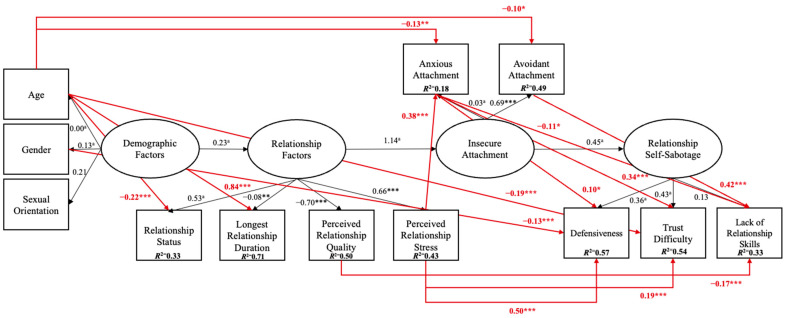
Standardised effects for Model 1. Notes: *a* = constrained parameter. *** ≤ 0.001; ** ≤ 0.005; * ≤ 0.05. Squares represent observable variables, and ellipses represent latent variables. Black arrows represent conceptual paths, while red arrows represent significant predicted paths.

Model 1 indicated a good fit (*χ*^2^_(37)_ = 46.963, *p* = 0.126; RMSEA = 0.025 [0.000, 0.044], *p* = 0.986; GFI = 0.983; CFI = 0.995; TLI = 0.990; SRMR = 0.032), as shown in Figure 4.

Inspection of Figure 4 and Table 1 shows that demographic factors, such as age, were a significant predictor of relationship status (−0.22, *p* ≤ 0.001), longest relationship duration (0.84, *p* ≤ 001), anxious attachment (−0.13, *p* ≤ 0.005), avoidant attachment (−0.10, *p* ≤ 0.05), and trust difficulty (−0.23, *p* ≤ 0.001). Further, gender was a significant predictor of defensiveness (−0.13, *p* ≤ 0.001). Regarding relationship factors, perceived relationship quality was a significant predictor of trust difficulty (−0.17, *p* ≤ 0.001), and perceived relationship stress was a significant predictor of anxious attachment (0.38, *p* ≤ 0.001), defensiveness (0.54, *p* ≤ 0.001), and trust difficulty (0.32, *p* ≤ 0.001). Regarding insecure attachment, anxious attachment was a significant predictor of defensiveness (0.10, *p* ≤ 0.05), trust difficulty (0.34, *p* ≤ 0.001), and relationship skills (−0.11, *p* ≤ 0.05). Avoidant attachment was a significant predictor of lack of relationship skills (0.42, *p* ≤ 0.001). Inspection of Table 1 also indicates an indirect effect from age to defensiveness (−0.01) and lack of relationship skills (−0.03), and from perceived relationship stress to lack of relationship skills (−0.04).

Altogether, the longest relationship duration explained the most variance in Final Model 1, with 71% (*R*^2^ = 0.71). Further, the following variables also explained model variance: relationship status (33%; *R*^2^ = 0.33), perceived relationship quality (50%; *R*^2^ = 0.50), perceived relationship stress (43%; *R*^2^ = 0.43), anxious attachment (18%; *R*^2^ = 0.18), avoidant attachment (49%; *R*^2^ = 0.49), defensiveness (57%; *R*^2^ = 0.57), trust difficulty (54%; *R*^2^ = 0.54), and lack of relationship skills (33%; *R*^2^ = 0.33).

Close inspection of the results from Model 1 (see Figure 4) also indicated that the relationship between relationship factors and insecure attachment is not linear (1.14). Although regression weights above 1 are valid (Jöreskog, 1967), they indicate a non-linear relationship and bi-directionality between the constructs, which is possibly a result of mediation effects (Fabrigar et al., 1999).

#### 3.2.2. Model 2

**Figure 5:**
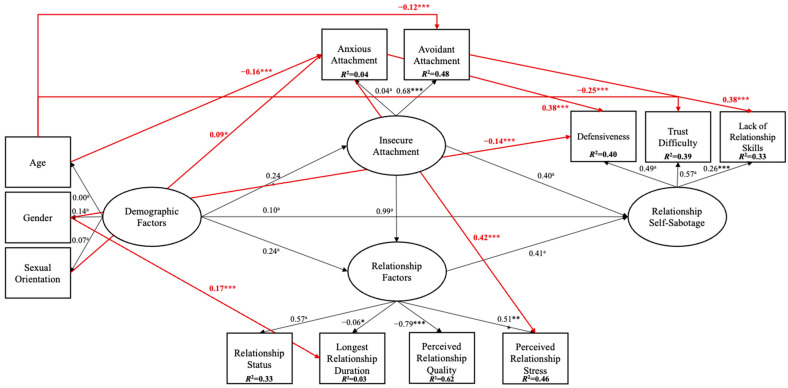
Standardised effects for Model 2. Notes: *a* = constrained parameter. *** ≤ 0.001; ** ≤ 0.005; * ≤ 0.05. Squares represent observable variables, and ellipses represent latent variables. Black arrows represent conceptual paths, while red arrows represent significant predicted paths.

Model 2 indicated a good fit (*χ*^2^_(37)_ = 48.144, *p* = 0.104; RMSEA = 0.026 [0.000, 0.045], *p* = 0.982; GFI = 0.983; CFI = 0.994; TLI = 0.989; SRMR = 0.031), as shown in Figure 5.

Inspection of Figure 5 and Table 2 shows that demographic factors, such as age (−0.16, *p* ≤ 0.001) and sexual orientation (0.09, *p* ≤ 0.05), were significant predictors of anxious attachment, and age was a significant predictor of avoidant attachment (−0.12, *p* ≤ 0.001) and trust difficulty (−0.25, *p* ≤ 0.001). Additionally, gender was a significant predictor of longest relationship duration (0.17, *p* ≤ 0.001) and defensiveness (−0.14, *p* ≤ 0.001). Regarding insecure attachment, anxious attachment was a significant predictor of perceived relationship stress (0.42, *p* ≤ 0.001) and defensiveness (0.38, *p* ≤ 0.001), and avoidant attachment was a significant predictor of lack of relationship skills (0.38, *p* ≤ 0.001). No significant paths were found between relationship factors and relationship sabotage in Model 2, which may be a result of mediation effects. Inspection of Table 2 also shows an indirect effect from age (−0.07) and gender (0.04) to perceived relationship stress, age (−0.06) and sexual orientation (0.03) to defensiveness, and age (−0.05) to lack of relationship skills.

Altogether, perceived relationship quality explained the most variance in Model 2, with 62% (*R*^2^ = 0.62). Further, the following variables also explained model variance: relationship status (33%; *R*^2^ = 0.33), longest relationship duration (3%; *R*^2^ = 0.03), perceived relationship stress (46%; *R*^2^ = 0.46), anxious attachment (4%; *R*^2^ = 0.04), avoidant attachment (48%; *R*^2^ = 0.48), defensiveness (40%; *R*^2^ = 0.40), trust difficulty (39%; *R*^2^ = 0.39), and lack of relationship skills (33%; *R*^2^ = 0.33).

#### 3.2.3. Model 3

**Figure 6:**
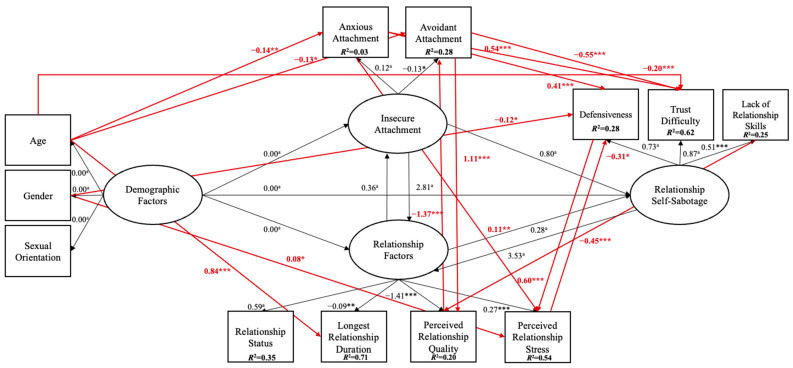
Standardised effects for Final Model 3. Notes: *a* = constrained parameter. *** ≤ 0.001; ** ≤ 0.005; * ≤ 0.05. Squares represent observable variables, and ellipses represent latent variables. Black arrows represent conceptual paths, while red arrows represent significant predicted paths.

Model 3 indicated a good fit (*χ*^2^_(36)_ = 39.206, *p* = 0.328; RMSEA = 0.014 [0.000, 0.038], *p* = 0.997; GFI = 0.985; CFI = 0.998; TLI = 0.997; SRMR = 0.036), as shown in Figure 6.

Inspection of Figure 6 and Table 3 shows that demographic factors, such as age, were a significant predictor of anxious attachment (−0.14, *p* ≤ 0.005), avoidant attachment (−0.5, *p* ≤ 0.05), longest relationship duration (0.84, *p* ≤ 0.001), and trust difficulty (−0.25, *p* ≤ 0.001), and gender was a significant predictor of perceived relationship stress (0.01, *p* ≤ 0.05) and defensiveness (−0.12, *p* ≤ 0.05). Regarding relationship factors, perceived relationship quality was a significant predictor of avoidant attachment (0.54, *p* ≤ 0.001), and perceived relationship stress was a significant predictor of defensiveness (−0.26, *p* ≤ 0.05). Regarding insecure attachment, anxious attachment was a significant predictor of perceived relationship stress (0.30, *p* ≤ 0.005), defensiveness (0.32, *p* ≤ 0.001), and trust difficulty (0.54, *p* ≤ 0.001), and avoidant attachment was a significant predictor of perceived relationship quality (0.44, *p* ≤ 0.001) and trust difficulty (−0.22, *p* ≤ 0.001). Finally, regarding relationship sabotage, defensiveness was a significant predictor of perceived relationship stress (0.51, *p* ≤ 0.001), and lack of relationship skills was a significant predictor of perceived relationship quality (−0.18, *p* ≤ 0.001).

Inspection of Table 3 also indicates an indirect effect from age to perceived relationship quality (−0.06), perceived relationship stress (−0.04), and defensiveness (−0.04); from perceived relationship quality to trust difficulty (0.30); and from lack of relationship skills to avoidant attachment (0.24) and trust difficulty (−0.13).

Altogether, the longest relationship duration explained the most variance in Model 3, with 71% (*R*^2^ = 0.71). Further, the following variables also explained model variance: relationship status (35%; *R*^2^ = 0.35), perceived relationship quality (20%; *R*^2^ = 0.20), perceived relationship stress (54%; *R*^2^ = 0.54), anxious attachment (3%; *R*^2^ = 0.03), avoidant attachment (28%; *R*^2^ = 0.28), defensiveness (28%; *R*^2^ = 0.28), trust difficulty (62%; *R*^2^ = 0.62), and lack of relationship skills (25%; *R*^2^ = 0.25).

Close inspection of the results from Model 3 showed a high regression weight between insecure attachment and relationship factors (2.81). The same was found between relationship sabotage and relationship factors (3.53). These results may be because of the existence of mediation effects.

## 4. Discussion

The results from the current study support the existing literature and go further to offer new ways of understanding relationship sabotage in intimate relationships. In accordance with Rusk and Rothbaum (2010), stress was found to be a significant predictor of anxious attachment (as shown in Model 1) and avoidant attachment (as shown in Model 3). Stress was also a significant predictor of defensiveness (as shown in Models 1 and 3) and trust difficulty (as shown in Model 1). However, as expected, another important relationship factor, perceived relationship quality, predicted insecure attachment. As shown in Model 3, low perceived relationship quality was a significant predictor of avoidant attachment. This finding indicates that focusing on ways to improve individuals’ perception of relationship quality, and in turn satisfaction, might be a better alternative to addressing insecure attachment, as opposed to minimising stress, which is not always possible. Also, this finding is supported by previous literature (e.g., Røsand et al., 2012), which shows that high relationship quality is a buffer to stress and can increase coping skills and improve self-esteem. Altogether, being in a relationship (especially one of high quality) is possibly a protective factor for insecure individuals seeking to avoid relationship sabotage. Further, relationship quality was found to be a significant predictor of relationship skills (as shown in Model 1). Being in a healthy relationship can also help foster relationship skills and subsequently lessen the effects of insecure attachment. This conclusion is in line with research conducted by Riggio et al. (2013) and Byl and Naydenova (2016), which suggested that willingness to learn to be a partner in a romantic engagement, as a product of self-efficacy, can be predictive of healthy relationship outcomes.

As expected, insecure attachment was also a significant predictor of relationship sabotage. Specifically, anxious attachment was a significant predictor of defensiveness (as shown in Models 1, 2, and 3), trust difficulty (as shown in Models 1 and 3), and relationship skills (as shown in Model 1). Avoidant attachment was a significant predictor of trust difficulty (as shown in Model 3) and lack of relationship skills (as shown in Models 1 and 2). Altogether, these findings suggest that the relationship between insecure attachment and relationship sabotage exists regardless of stress. However, stress can strengthen the relationship between insecure attachment and relationship sabotage. Altogether these findings reinforce the notion that an individual’s perception of what is happening for them in the relationship has the most significance, above and beyond what is in fact happening in reality.

Further, results support the knowledge that avoidant attachment is a stronger predictor of sabotage, as evidenced by the R-squared values across all models. This is expected given our previous investigations, which indicated that individuals with an avoidant attachment style are more likely to engage in relationship self-defeating attitudes and behaviours (Peel & Caltabiano, 2020). But, in the past, the literature has mostly focused on anxious attachment and its representative traits (e.g., rejection sensitivity). Accordingly, much work has been carried out to show how individuals’ expectations of rejection often lead to relationship break-ups. An example of this is research conducted by Elliot and Reis (2003), which suggested that individuals with anxious attachment are more prone to avoidance goals. However, Locke (2008) clarified that both individuals with anxious and avoidant attachment resort to avoidance goals—the difference is that individuals with anxious attachment tend to experience approach goals as well, which individuals with avoidant attachment do not. As an example, Meyer et al. (2005) found that women with anxious attachment displayed great emotional distress and impulses to express both approaching behaviours (e.g., to engage with the partner) and avoidance behaviours (e.g., to seek distance from the partner). In contrast, avoidant attachment only predicted avoidance behaviours, which would be more aligned with relationship sabotage. These findings suggest that avoidant attachment is a key characteristic of relationship sabotage.

Interestingly, results also suggested that being in a long-term relationship can be a protective factor against defensiveness for individuals with low anxiety. This indicates that over time maladaptive attitudes and behaviours can be modified, especially in a context where one might feel secure in their relationship. Results also showed a negative relationship between avoidant attachment and trust difficulty (as shown in Model 3), which suggests that less avoidance can lead to more trust difficulty. A plausible explanation of this result is based on the relationship between anxious attachment and trust difficulty. It is well known that individuals with anxious attachment tend to have difficulty with trust, as evidenced in the previous literature and the current research. In addition, it is understood that individuals who are low in avoidance are not necessarily secure. In turn, they can be anxious and untrusting. To support this argument, previous literature (e.g., Shaver & Fraley, 2004) has established that the opposite of dismissive avoidance is anxious preoccupation, which would explain the negative relationship between avoidance and trust difficulty. Similarly, a negative relationship between perceived relationship stress and defensiveness (as shown in Model 3) indicates that involvement in the relationship (or relational investment), which is a concept not directly tested, could be central to understanding sabotage (Fletcher & Simpson, 2000; Le et al., 2010).

As hypothesised, insecure attachment was also found to be a predictor of relationship factors. Specifically, anxious attachment was a significant predictor of perceived relationship stress (as shown in Models 2 and 3). In addition, avoidant attachment was a significant predictor of relationship quality (as shown in Model 3). In turn, relationship sabotage was also a predictor of relationship factors. Specifically, defensiveness was a significant predictor of perceived relationship stress (as shown in Model 3), and relationship skill was a significant predictor of relationship quality (as shown in Model 3). These findings suggest that insecure attachment and relationship sabotage tendencies will influence how people perceive their relationship overall, which in turn will affect how they respond to difficult times (and stress) in the relationship, as it is well established. Nevertheless, results overall are encouraging and suggest that insecure attachment is not necessarily a ‘death sentence’, inevitably leading to relationship sabotage. Specifically, and as aforementioned, dealing with defensiveness and trust issues appears to be a key protective factor to help insecure individuals attain a high-quality relationship.

In conclusion, all models displayed interesting results. However, the alternative models to Rusk and Rothbaum’s (2010) linear premise showed a more complete picture to represent the true nature of intimate relationships, which is not linear and clear. Models 2 and 3 highlighted how different indirect paths could change and reshape the fate of relationships. However, Model 3 was the only model to show a significant path from relationship sabotage to relationship factors. This is because Model 3 is a non-recursive model showing reciprocal effects between insecure attachment and relationship factors and between relationship factors and relationship sabotage.

### 4.1. Limitations and Future Directions

It is important to note that non-linear models do possess limitations. Spiess and Neumeyer (2010) argued that regression weights and the R-squared statistic are not the best measures to understand the results from non-linear models. They suggested using Akaike information criterion and Bayesian information criterion as an alternative, which could be explored in future studies. Nevertheless, results interpreted using regression weights and R-squared are still largely acceptable and not invalid (Kline, 2016). Yet, it would be good practice to exercise caution when interpreting the estimation values provided in the current study, which involved cross-sectional data. There is also the possibility for confounding conclusions regarding trust difficulty when interpreting results involving the PRQCI-SF (Fletcher et al., 2000) and the RSS (Peel & Caltabiano, 2021). Future studies would also benefit from retesting the proposed models with different samples. Additionally, future studies should explore individual differences—such as gender, age, and sexual orientation. Nevertheless, support is already shown in the existing mean differences between the age, gender, and sexual orientation groups. Cultural differences are also expected to come into effect. Another recommendation for future studies is to investigate mediation effects, which may further explain ways to break the pattern of relationship sabotage. More specifically, given the results from the full models, we expect perceived relationship quality might mediate the relationship between anxious attachment and lack of relationship skills. Also, avoidant attachment might mediate the relationships between perceived relationship stress and lack of relationship skills and between perceived relationship quality and trust difficulty.

### 4.2. Study Strengths and Implications

This paper has detailed a preliminary evaluation of a theoretical framework for relationship sabotage. Attachment and goal-orientation theories are integrated to support the conceptualisation of a phenomenon that up until recently was not well understood and empirically defined. The measure for relationship sabotage has now been validated across studies and cultures, and theory development has shaped a unique space to understand sabotage in intimate relationships as a persistent phenomenon that sees individuals employ self-defeating attitudes and behaviours that prevent them from establishing and maintaining fulfilling intimate engagements. This study also reinforces the importance of identifying and targeting these maladaptive relational patterns in therapy with individuals and couples, as previously established (see Peel et al., 2019). Understanding these maladaptive attitudes and behaviours as modifiable can encourage individuals to break the cycle of sabotage across relationships and has the potential to foster healthier relationship models for young people and those learning about intimate engagements.

## 5. Conclusions

Overall, the results from this study show that the best model for relationship sabotage is not linear. The way people arrive at relationship sabotage is best demonstrated in a circular manner. While insecure attachment leads to self-sabotage, sabotaging relationships reinforces existing insecure attachment styles or establishes new vulnerable styles. Further, self-sabotaging tendencies influence how people perceive quality and stress in their relationship, which means that individuals’ own attitudes and behaviours might be preventing them from starting and maintaining fulfilling intimate relationships. Thus, investing in learning and fostering healthy relationship skills can lessen the effects of insecure attachment. Lastly, dealing with defensiveness and trust issues, specifically, appears to be a key protective factor to help insecure individuals attain high-quality relationships.

## Figures and Tables

**Table 1:** Model 1: standardised effects of exogenous variables on endogenous variables.

	Endogenous Variables																										
	Relationship Status			Longest Relationship Duration			Perceived Relationship Quality			Perceived Relationship Stress			Anxious Attachment			Avoidant Attachment			Defensiveness			Trust Difficulty			Lack of Relationship Skills		
	Effects (*R*^2^)			Effects (*R*^2^)			Effects (*R*^2^)			Effects (*R*^2^)			Effects (*R*^2^)			Effects (*R*^2^)			Effects (*R*^2^)			Effects (*R*^2^)			Effects (*R*^2^)		
**Exogenous Variables**	D	I	T	D	I	T	D	I	T	D	I	T	D	I	T	D	I	T	D	I	T	D	I	T	D	I	T
**Age**	−0.22 ***	0.00	−0.22	0.84 ***	0.00	0.84	0.00	0.00	0.00	0.00	0.00	0.00	−0.13 **	0.00	−0.13	−0.10 *	0.00	−0.10	0.00	−0.01	−0.01	−0.19 ***	−0.04	−0.23	0.00	−0.03	−0.03
**Gender**	0.00	0.00	0.00	0.00	0.00	0.00	0.00	0.00	0.00	0.00	0.00	0.00	0.00	0.00	0.00	0.00	0.00	0.00	−0.13 ***	0.00	−0.13	0.00	0.00	0.00	0.00	0.00	0.00
**Sexual Orientation**	0.000	0.00	0.00	0.00	0.00	0.00	0.00	0.00	0.00	0.00	0.00	0.00	0.00	0.00	0.00	0.00	0.00	0.00	0.00	0.00	0.00	0.00	0.00	0.00	0.00	0.00	0.00
**Relationship Status**	–			0.00	0.00	0.00	0.00	0.00	0.00	0.00	0.00	0.00	0.00	0.00	0.00	0.00	0.00	0.00	0.00	0.00	0.00	0.00	0.00	0.00	0.00	0.00	0.00
**Longest Relationship Duration**	0.00	0.00	0.00	–			0.00	0.00	0.00	0.00	0.00	0.00	0.00	0.00	0.00	0.00	0.00	0.00	0.00	0.00	0.00	0.00	0.00	0.00	0.00	0.00	0.00
**Perceived Relationship Quality**	0.00	0.00	0.00	0.00	0.00	0.00	–			0.00	0.00	0.00	0.00	0.00	0.00	0.00	0.00	0.00	0.00	0.00	0.00	0.00	0.00	0.00	−0.17 ***	0.00	−0.17
**Perceived Relationship Stress**	0.00	0.00	0.00	0.00	0.00	0.00	0.00	0.00	0.00	–			0.38 ***	0.00	0.38	0.00	0.00	0.00	0.50 ***	0.04	0.54	0.19 ***	0.13	0.32	0.00	−0.04	−0.04
**Anxious Attachment**	0.00	0.00	0.00	0.00	0.00	0.00	0.00	0.00	0.00	0.00	0.00	0.00	–			0.00	0.00	0.00	0.10 *	0.00	0.10	0.34 ***	0.00	0.34	−0.11 *	0.00	−0.11
**Avoidant Attachment**	0.00	0.00	0.00	0.00	0.00	0.00	0.00	0.00	0.00	0.00	0.00	0.00	0.00	0.00	0.00	–			0.00	0.00	0.00	0.00	0.00	0.00	0.42 ***	0.00	0.42

Notes: *** ≤ 0.001; ** ≤ 0.005; * ≤ 0.05.

**Table 2:** Model 2: standardised effects of exogenous variables on endogenous variables.

	Endogenous Variables																										
	Anxious Attachment			Avoidant Attachment			Relationship Status			Longest Relationship Duration			Perceived Relationship Quality			Perceived Relationship Stress			Defensiveness			Trust Difficulty			Lack of Relationship Skills		
	Effects (*R*^2^)			Effects (*R*^2^)			Effects (*R*^2^)			Effects (*R*^2^)			Effects (*R*^2^)			Effects (*R*^2^)			Effects (*R*^2^)			Effects (*R*^2^)			Effects (*R*^2^)		
**Exogenous Variables**	D	I	T	D	I	T	D	I	T	D	I	T	D	I	T	D	I	T	D	I	T	D	I	T	D	I	T
**Age**	**−** 0.16 ***	0.00	−0.16	**−** 0.12 **	0.00	−0.12	0.00	0.00	0.00	0.00	0.00	0.00	0.00	0.00	0.00	0.00	−0.07	−0.07	0.00	−0.06	−0.06	**−** 0.25 ***	0.00	−0.25	0.00	−0.05	−0.05
**Gender**	0.00	0.00	0.00	0.00	0.00	0.00	0.00	0.00	0.00	**−** 0.17 ***	0.00	−0.17	0.00	0.00	0.00	0.00	0.04	0.04	**−** 0.14 ***	0.00	−0.14	0.00	0.00	0.00	0.00	0.00	0.00
**Sexual Orientation**	0.09 *	0.00	0.09	0.00	0.00	0.00	0.00	0.00	0.00	0.00	0.00	0.00	0.00	0.00	0.00	0.00	0.00	0.00	0.00	0.03	0.03	0.00	0.00	0.00	0.00	0.00	0.00
**Anxious Attachment**	–			0.00	0.00	0.00	0.00	0.00	0.00	0.00	0.00	0.00	0.00	0.00	0.00	0.42 ***	0.00	0.42	0.38 ***	0.00	0.38	0.00	0.00	0.00	0.00	0.00	0.00
**Avoidant Attachment**	0.00		0.00	–			0.00	0.00	0.00	0.00	0.00	0.00	0.00	0.00	0.00	0.00	0.00	0.00	0.00	0.00	0.00	0.00	0.00	0.00	0.38 ***	0.00	0.38
**Relationship Status**	0.00	0.00	0.00	0.00	0.00	0.00	0.00			0.00	0.00	0.00	0.00	0.00	0.00	0.00	0.00	0.00	0.00	0.00	0.00	0.00	0.00	0.00	0.00	0.00	0.00
**Longest Relationship Duration**	0.00	0.00	0.00	0.00	0.00	0.00	0.00	0.00	0.00	**–**			0.00	0.00	0.00	0.00	0.00	0.00	0.00	0.00	0.00	0.00	0.00	0.00	0.00	0.00	0.00
**Perceived Relationship Quality**	0.00	0.00	0.00	0.00	0.00	0.00	0.00	0.00	0.00	0.00	0.00	0.00	–			0.00	0.00	0.00	0.00	0.00	0.00	0.00	0.00	0.00	0.00	0.00	0.00
**Perceived Relationship Stress**	0.00	0.00	0.00	0.00	0.00	0.00	0.00	0.00	0.00	0.00	0.00	0.00	0.00	0.00	0.00	–			0.00	0.00	0.00	0.00	0.00	0.00	0.00	0.00	0.00

Notes: *** ≤ 0.001; ** ≤ 0.005; * ≤ 0.05.

**Table 3:** Model 3: standardised effects of exogenous variables on endogenous variables.

	Endogenous Variables																										
	Anxious Attachment			Avoidant Attachment			Relationship Status			Longest Relationship Duration			Perceived Relationship Quality			Perceived Relationship Stress			Defensiveness			Trust Difficulty			Lack of Relationship Skills		
	Effects (*R*^2^)			Effects (*R*^2^)			Effects (*R*^2^)			Effects (*R*^2^)			Effects (*R*^2^)			Effects (*R*^2^)			Effects (*R*^2^)			Effects (*R*^2^)			Effects (*R*^2^)		
**Exogenous Variables**	D	I	T	D	I	T	D	I	T	D	I	T	D	I	T	D	I	T	D	I	T	D	I	T	D	I	T
**Age**	−0.14 **	0.00	**−0.14**	−0.13 *	0.08	−0.05	0.00	0.00	0.00	0.84 ***	0.00	0.84	0.00	−0.06	−0.06	0.00	−0.04	−0.04	0.00	−0.04	−0.04	−0.20 ***	−0.05	−0.25	0.00	0.00	0.00
**Gender**	0.00	0.00	0.00	0.00	0.00	0.00	0.00	0.00	0.00	0.00	0.00	0.00	0.00	0.00	0.00	0.08 *	−0.07	0.01	−0.12 *	0.00	−0.12	0.00	0.00	0.00	0.00	0.00	0.00
**Sexual Orientation**	0.00	0.00	0.00	0.00	0.00	0.00	0.00	0.00	0.00	0.00	0.00	0.00	0.00	0.00	0.00	0.00	0.00	0.00	0.00	0.00	0.00	0.00	0.00	0.00	0.00	0.00	0.00
**Anxious Attachment**	–			0.00	0.00	0.00	0.00	0.00	0.00	0.00	0.00	0.00	0.00	0.00	0.00	0.11 **	0.19	0.30	0.41 ***	−0.09	0.32	0.54 ***	0.00	0.54	0.00	0.00	0.00
**Avoidant Attachment**	0.00	0.00	0.00	–			0.00	0.00	0.00	0.00	0.00	0.00	10.11 ***	−0.67	0.44	0.00	0.00	0.00	0.00	0.00	0.00	−0.55 ***	0.33	−0.22	0.00	0.00	0.00
**Perceived Relationship Quality**	0.00	0.00	0.00	−10.37 ***	0.83	−0.54	0.00	0.00	0.00	0.00	0.00	0.00	–			0.00	0.00	0.00	0.00	0.00	0.00	0.00	0.30	0.30	0.00	0.00	0.00
**Perceived Relationship Stress**	0.00	0.00	0.00	0.00	0.00	0.00	0.00	0.00	0.00	0.00	0.00	0.00	0.00	0.00	0.00	–			−0.31 *	0.05	−0.26	0.00	0.00	0.00	0.00	0.00	0.00
**Defensiveness**	0.00	0.00	0.00	0.00	0.00	0.00	0.00	0.00	0.00	0.00	0.00	0.00	0.00	0.00	0.00	0.60 ***	−0.09	0.51	–			0.00	0.00	0.00	0.00	0.00	0.00
**Trust Difficulty**	0.00	0.00	0.00	0.00	0.00	0.00	0.00	0.00	0.00	0.00	0.00	0.00	0.00	0.00	0.00	0.00	0.00	0.00	0.00	0.00	0.00	–			0.00	0.00	0.00
**Lack of Relationship Skills**	0.00	0.00	0.00	0.00	0.24	0.24	0.00	0.00	0.00	0.00	0.00	0.00	−0.45 ***	0.27	−0.18	0.00	0.00	0.00	0.00	0.00	0.00	0.00	−0.13	−0.13	–		

Notes: *** ≤ 0.001; ** ≤ 0.005; * ≤ 0.05.

## Data Availability

The datasets used and/or analysed during the current study are available from the corresponding author on reasonable request.
